# Performance of a Multigene Genomic Classifier in Thyroid Nodules With Indeterminate Cytology: A Prospective Blinded Multicenter Study

**DOI:** 10.1001/jamaoncol.2018.4616

**Published:** 2018-11-08

**Authors:** David L. Steward, Sally E. Carty, Rebecca S. Sippel, Samantha Peiling Yang, Julie A. Sosa, Jennifer A. Sipos, James J. Figge, Susan Mandel, Bryan R. Haugen, Kenneth D. Burman, Zubair W. Baloch, Ricardo V. Lloyd, Raja R. Seethala, William E. Gooding, Simion I. Chiosea, Cristiane Gomes-Lima, Robert L. Ferris, Jessica M. Folek, Raheela A. Khawaja, Priya Kundra, Kwok Seng Loh, Carrie B. Marshall, Sarah Mayson, Kelly L. McCoy, Min En Nga, Kee Yuan Ngiam, Marina N. Nikiforova, Jennifer L. Poehls, Matthew D. Ringel, Huaitao Yang, Linwah Yip, Yuri E. Nikiforov

**Affiliations:** 1 Department of Otolaryngology, Head and Neck Surgery, University of Cincinnati Medical Center, Cincinnati, Ohio; 2 Division of Endocrine Surgery, University of Pittsburgh, Pittsburgh, Pennslyvania; 3 Division of Endocrine Surgery, University of Wisconsin, Madison; 4 Endocrinology Division, Department of Medicine, National University Hospital, Singapore, Singapore; 5 Section of Endocrine Surgery, Department of Surgery, Duke Cancer Institute and Duke Clinical Research Institute, Duke University, Durham, North Carolina; 6 Department of Surgery, University of California, San Francisco; 7 Division of Endocrinology, Diabetes, and Metabolism, The Ohio State University School of Medicine, Columbus; 8 Diabetes & Endocrine Care, St Peter's Health Partners, Rensselaer, New York; 9 Division of Endocrinology, Diabetes and Metabolism, Perelman School of Medicine, University of Pennsylvania, Philadelphia; 10 Division of Endocrinology, Metabolism and Diabetes, University of Colorado School of Medicine, Aurora; 11 Department of Medicine, Endocrinology Section, MedStar Washington Hospital Center, Washington, DC; 12 Department of Pathology and Laboratory Medicine, Perelman School of Medicine, University of Pennsylvania, Philadelphia; 13 Department of Pathology and Laboratory Medicine, University of Wisconsin, Madison; 14 Department of Pathology, University of Pittsburgh, Pittsburgh, Pennsylvania; 15 Biostatistics Facility, UPMC Hillman Cancer Center, Pittsburgh, Pennsylvania; 16 Departments of Otolaryngology and Immunology, UPMC Hillman Cancer Center, Pittsburgh, Pennsylvania; 17 Department of Otolaryngology–Head and Neck Surgery, National University Hospital, Singapore; 18 Department of Pathology, University of Colorado School of Medicine, Aurora, Colorado; 19 Department of Pathology, National University Hospital, Singapore; 20 Department of General Surgery, University Surgical Cluster, National University Hospital, Singapore; 21 Division of Endocrinology, Diabetes & Metabolism, University of Wisconsin, Madison; 22 Department Pathology, University of Cincinnati Medical Center, Cincinnati, Ohio

## Abstract

**Importance:**

Approximately 20% of fine-needle aspirations (FNA) of thyroid nodules have indeterminate cytology, most frequently Bethesda category III or IV. Diagnostic surgeries can be avoided for these patients if the nodules are reliably diagnosed as benign without surgery.

**Objective:**

To determine the diagnostic accuracy of a multigene classifier (GC) test (ThyroSeq v3) for cytologically indeterminate thyroid nodules.

**Design, Setting, and Participants:**

Prospective, blinded cohort study conducted at 10 medical centers, with 782 patients with 1013 nodules enrolled. Eligibility criteria were met in 256 patients with 286 nodules; central pathology review was performed on 274 nodules.

**Interventions:**

A total of 286 FNA samples from thyroid nodules underwent molecular analysis using the multigene GC (ThyroSeq v3).

**Main Outcomes and Measures:**

The primary outcome was diagnostic accuracy of the test for thyroid nodules with Bethesda III and IV cytology. The secondary outcome was prediction of cancer by specific genetic alterations in Bethesda III to V nodules.

**Results:**

Of the 286 cytologically indeterminate nodules, 206 (72%) were benign, 69 (24%) malignant, and 11 (4%) noninvasive follicular thyroid neoplasms with papillary-like nuclei (NIFTP). A total of 257 (90%) nodules (154 Bethesda III, 93 Bethesda IV, and 10 Bethesda V) had informative GC analysis, with 61% classified as negative and 39% as positive. In Bethesda III and IV nodules combined, the test demonstrated a 94% (95% CI, 86%-98%) sensitivity and 82% (95% CI, 75%-87%) specificity. With a cancer/NIFTP prevalence of 28%, the negative predictive value (NPV) was 97% (95% CI, 93%-99%) and the positive predictive value (PPV) was 66% (95% CI, 56%-75%). The observed 3% false-negative rate was similar to that of benign cytology, and the missed cancers were all low-risk tumors. Among nodules testing positive, specific groups of genetic alterations had cancer probabilities varying from 59% to 100%.

**Conclusions and Relevance:**

In this prospective, blinded, multicenter study, the multigene GC test demonstrated a high sensitivity/NPV and reasonably high specificity/PPV, which may obviate diagnostic surgery in up to 61% of patients with Bethesda III to IV indeterminate nodules, and up to 82% of all benign nodules with indeterminate cytology. Information on specific genetic alterations obtained from FNA may help inform individualized treatment of patients with a positive test result.

## Key Points

**Question:**

Can the diagnosis of benign disease or cancer in thyroid nodules with indeterminate cytology be established by molecular testing instead of diagnostic surgery?

**Findings:**

This prospective, blinded, multicenter cohort study of a multigene genomic classifier (ThyroSeq v3) test included 257 indeterminate cytology thyroid nodules with informative test results. It demonstrated a high sensitivity (94%) and reasonably high specificity (82%), with 61% of the nodules yielding a negative test result and only 3% residual cancer risk in these nodules.

**Meanings:**

Up to 61% of patients with indeterminate cytology thyroid nodules may avoid diagnostic surgery by undergoing multigene genomic classifier testing.

## Teaser

This prospective study examines the diagnostic accuracy of a multigene genomic classifier test for diagnosis of benign disease or cancer in thyroid nodules with indeterminate cytology.

## Introduction

Thyroid nodules are common, with as many as two-thirds of adults harboring nodules detectable by ultrasound and about 5% by palpation.^1,2^ Ultrasound has proven useful in estimating the likelihood of malignant tumors and selecting nodules for fine-needle aspiration (FNA) biopsy.^3,4,5,6,7^ More than 600 000 thyroid FNAs are performed every year in the United States alone, and the number has been increasing annually by 16%.^8,9^ Thyroid FNA cytology can accurately classify most nodules as benign and a minority as malignant, but the results remain indeterminate in about 20% (range, 10%-38%) of nodules when cytological features lack specific characteristics needed for a definitive diagnosis.^10^ Further, the proportion of indeterminate cytology results appears to be rising.^11^

The Bethesda System for Reporting Thyroid Cytopathology^12,13^ includes 2 common categories of indeterminate cytology, atypical or follicular lesion of undetermined significance (Bethesda category III) and follicular neoplasm/suspicious for follicular (or Hürthle cell) neoplasm (Bethesda category IV), each accounting for approximately 10% of all thyroid FNA results.^10^ The observed rates of cancer in these categories vary widely by institution, ranging from 6% to 48% for Bethesda III and 14% to 34% for Bethesda IV,^10^ which poses diagnostic uncertainty that greatly confounds patient treatment, often resulting in repeat FNA and/or unnecessary diagnostic surgery.^3^ Another cytologic category generally considered as indeterminate is suspicious for malignancy (Bethesda category V), which comprises 2% to 3% of all FNAs.^10,12,13^ The probability of cancer in these nodules is much higher, 53% to 97%,^10^ with surgery indicated in most cases, although the extent of surgery (thyroid lobectomy or total thyroidectomy with possible elective central lymph node dissection) could be informed by a more precise cancer probability assessment.^3^

Most thyroid cancers are well differentiated and have an indolent clinical course and low mortality. As a result, limited surgery and lower intensity postsurgical treatment and surveillance may be considered for cancers with low to intermediate risk for recurrence.^3^ In addition, the histologic assessment (the gold standard for cytology) of benign vs malignant thyroid nodules is in transition with both intraobserved and interobserved variability rates that can be high^14^ and a recent change in nomenclature for the noninvasive encapsulated follicular variant of papillary thyroid cancer.^15^ Now variably considered as nonmalignant, premalignant, or possibly carcinoma in situ, and redefined as a noninvasive follicular thyroid neoplasm with papillary-like nuclear features (NIFTP),^15,16^ these tumors require surgery for diagnosis and treatment, but can usually be adequately treated by lobectomy.^17,18^ Furthermore, thyroid cancers driven by distinct mutations (most commonly *BRAF* V600E or *RAS*) differ with respect to their pathologic and clinical properties,^19,20^ and accumulation of additional mutations such as *TERT* may identify thyroid cancers with the highest risk for tumor recurrence and disease-specific mortality.^21,22^

Over the past decade, molecular testing of thyroid nodules was developed to improve diagnostic accuracy of FNA cytology.^23,24^ The initial small gene mutation panels offered high PPV for cancer detection but lacked sufficiently high NPV to reliably exclude malignant disease in test-negative samples.^25,26^ More advanced molecular tests were subsequently developed using gene expression profiling, broader panels of mutational markers, or combinations of different markers.^27,28,29,30,31^ Overall, they offered a significantly improved sensitivity and NPV. However, they suffer from either relatively low specificity and PPV, particularly for certain types of thyroid cancer, such as Hürthle cell tumors, limited clinical validation, and/or lack of reporting specific molecular information for more refined cancer risk assessment.

Recently, a new 112-gene test was developed (ThyroSeq v3 Genomic Classifier [GC]) to include a broad range of thyroid cancer-related point mutations, gene fusions, copy number alterations and gene expression alterations with the goals of achieving both high sensitivity and specificity in detecting all types of thyroid cancer and providing detailed genomic information on the nodules sampled by FNA biopsy.^32^ This prospective, blinded, multicenter clinical validation study was undertaken to assess the diagnostic performance of this GC test in cytologically indeterminate thyroid nodules.

## Methods

### Study Population

Patients eligible for this study were aged 18 years or older, had 1 or more thyroid nodules, underwent a routine FNA procedure to collect samples for cytological examination, and agreed to provide material for molecular analysis. After FNA cytology was reported, only those patients who had at least 1 nodule that yielded a cytologic diagnosis of Bethesda III, IV, or V and underwent thyroid surgery to remove 1 or more nodules were included in the study.

### Study Design and Sample Collection

**Figure 1.**
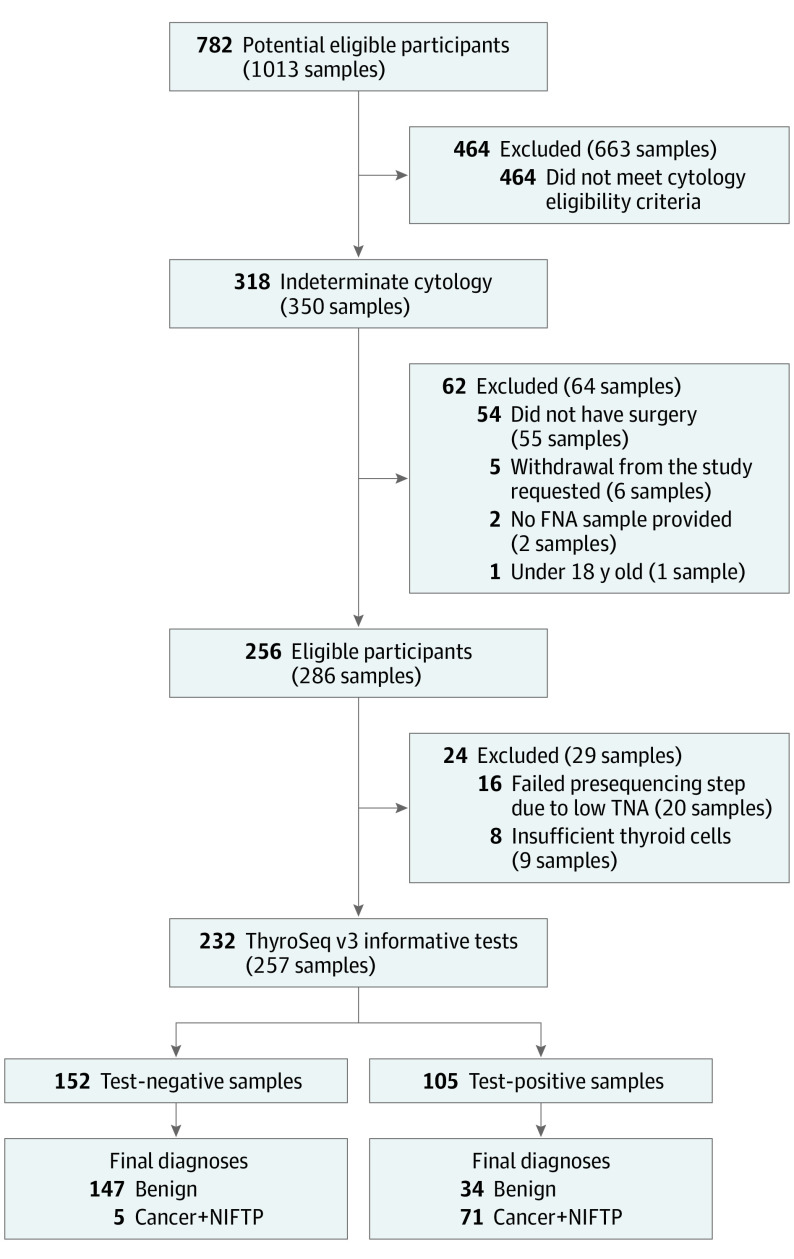
Recruitment and Exclusion of Patients and Samples in the Study FNA indicates fine-needle aspiration; TNA, total nucleic acids; NIFTP, noninvasive follicular thyroid neoplasm with papillary-like nuclear features.

This prospective cohort study recruited 782 patients with 1013 thyroid nodules clinically evaluated at 10 sites, 9 in the United States and 1 in Singapore, between January 2015 and December 2016. All FNA were performed using a 22g, 25g, or 27g needle depending on institutional practice. Samples were collected for molecular analysis by either (1) rinsing the residual material in the aspiration needle from all passes or (2) collecting a dedicated pass into a preservative solution tube (ThyroSeq*Preserve*) and stored at −20°C. Samples from nodules diagnosed as Bethesda III, IV, or V with surgical follow-up were retained as eligible and shipped to the University of Pittsburgh Medical Center (UPMC) for GC testing. Application of the eligibility criteria resulted in 256 patients with thyroid nodules that yielded 286 FNA samples available for molecular analysis (Figure 1). Central pathology review was performed on 274 (96%) nodules by a panel of expert thyroid pathologists (eMethods 1 in the Supplement).

The study was double-blinded; neither cytologists nor pathologists were aware of molecular analysis results and none of the personnel involved in performing molecular analysis were aware of cytology and histopathology results. The study was approved by the institutional review boards or ethics committees of all participating study sites. Written informed consent was obtained and patients were not compensated for participation. The study protocol is available (ClinicalTrials.gov identifier: NCT02352766).

### Molecular Analysis

The ThyroSeq v3 GC is a targeted next-generation sequencing test that interrogates selected regions of 112 thyroid cancer-related genes for point mutations, insertions/deletions, gene fusions, copy number alterations, or gene expression alterations.^32^ The assay was performed at the UPMC Molecular and Genomic Pathology laboratory.^32^ The genomic classifier was applied to assign a value to each detected genetic alteration based on the strength of association with malignancy: 0 (no association with cancer), 1 (low cancer probability), or 2 (high cancer probability). A GC score calculated for each sample is a sum of individual values of all detected alterations, with GC scores 0 and 1 accepted as test negative (score 1 commercially reported as currently negative) and scores 2 and above as test positive.^32^

### Study Outcomes

The primary outcome was the sensitivity, specificity, NPV, and PPV of the multigene GC to predict the histopathologic diagnosis of benign nodule vs cancer/NIFTP in indeterminate thyroid nodules with Bethesda III and IV cytology. In data analysis, NIFTP was grouped together with cancer because it also represents a tumor type that requires surgery based on current practice guidelines.^17,18^ The secondary outcome was the prediction of cancer/NIFTP by specific genetic alterations in Bethesda III, IV, and V cytology nodules.

### Statistical Analysis

For the primary and secondary outcomes, the test sensitivity, specificity, PPV, and NPV with 95% Wilson confidence intervals were calculated^33^ for individual nodules using the consensus diagnosis of central pathology as the reference standard. Using observed sensitivity and specificity, hypothetical positive and negative predictive value curves were calculated over the entire range (0%-100%) of possible disease prevalence. Among patients with nodules yielding indeterminate cytology, baseline characteristics of the included and excluded patients were compared using the Wilcoxon test and Fisher exact test. Statistical analysis was conducted with the R software package (version 3.4.2, R Foundation).^34^ Sample size justification and the programming code used to generate results are described in eMethods 2 in the Supplement.

## Results

### Patients and Nodules

Of the 256 eligible patients, 202 were female (79%), with a median age of 53 years (range, 18-90 years); biopsied nodules had a median size of 2.4 cm (range, 0.5-7 cm). Among the 286 eligible samples, FNA cytology diagnosis was Bethesda III in 172, Bethesda IV in 101, and Bethesda V in 13 cases. Based on the results of central pathology review, 206 nodules (72%) were classified as benign, 69 (24%) as malignant, and 11 (4%) as NIFTP. The prevalence of conditions requiring surgery, ie, cancer and NIFTP, was 28% in the entire cohort, ranging from 9% to 60% among study sites (eTable 2 in the Supplement).

### Molecular Analysis

Of 286 samples subjected to molecular analysis, 20 (7%) failed a presequencing step owing to low total nucleic acid quantity reflecting low sample cellularity, and 9 (3%) were inadequate on postsequencing analysis because the expression of thyroid cell markers was below the established acceptable level.^32^ Thus, 257 (90%) samples from 232 patients were informative for molecular analysis comprising the final study set. It included samples from 154 Bethesda III, 93 Bethesda IV, and 10 Bethesda V nodules. Molecular analysis yielded a negative test result in 152 (59%) samples and a positive result in 105 (41%) samples (eTable 4 in the Supplement). Among all 318 patients with indeterminate cytology, baseline characteristics of the included and excluded (Figure 1) patients and nodules were similar (eTable 3 in the Supplement).

### Overall Test Performance

The primary outcome of this study was the accurate separation of histopathological benign nodules from cancer and NIFTP in samples with Bethesda III and IV cytology. Table 1 summarizes the test sensitivity, specificity, NPV, and PPV in these cytologic groups. Overall, in Bethesda III and IV nodules, a negative or benign call rate was 61%.

**Table 1.** Performance of the Genomic Classifier Test in Cytologically Indeterminate Thyroid Nodules

**Performance in Bethesda III nodules (n = 154; disease prevalence 23%)**			
Result	Cancer+NIFTP (n = 35)	Benign (n = 119)	Test performance, % (95% CI)
Positive	32	18	Sensitivity, 91 (77-97) Specificity, 85 (77-90) NPV, 97 (92-99) PPV, 64 (50-77)
Negative	3	101	
**Performance in Bethesda IV nodules (n = 93; disease prevalence 35%)**			
Result	Cancer+NIFTP (n = 33)	Benign (n = 60)	Test performance, % (95% CI)
Positive	32	15	Sensitivity, 97(85-100) Specificity, 75(63-84) NPV, 98(89-100) PPV, 68 (54-80)
Negative	1	45	
**Performance in Bethesda III and IV nodules (n = 247; disease prevalence 28%)**			
Result	Cancer+NIFTP (n = 68)	Benign (n = 179)	Result
Positive	64	33	Sensitivity, 94 (86-98) Specificity, 82 (75-87) NPV, 97 (93-99) PPV, 66 (56-75)
Negative	4	146	
**Performance Across the Entire Cohort (n = 257; Disease Prevalence 30%)**			
Result	Cancer+NIFTP (n = 76)	Benign (n = 181)	Test performance, % (95% CI)
Positive	71	34	Sensitivity, 93 (86-97) Specificity, 81 (75-86) NPV, 97 (93-99) PPV, 68 (58-76)
Negative	5	147	

Abbreviations: NIFTP, Noninvasive follicular thyroid neoplasm with papillary-like nuclear features; NPV, negative predictive value; PPV, positive predictive value.

Test performance in specific histopathologic types of thyroid nodules is presented in Table 2. Among nodules found to be benign after surgery, the test correctly classified as negative 84 of 95 (88%) hyperplastic follicular cell nodules, 5 of 5 (100%) hyperplastic Hürthle cell nodules, 37 of 47 (79%) follicular adenomas, and 21 of 34 (62%) Hürthle cell adenomas. The GC scores in these benign nodules were 0 in 86% and 1 in 14% (eFigure in the Supplement). In a subgroup of histologically benign nodules with Bethesda III-IV cytology, 146 of 179 (82%) were classified as negative.

**Table 2.** Test Performance in Specific Histopathologic Types of Thyroid Lesions

Histopathologic Diagnosis	Nodules, No. (%)	Test		Correctly Classified, % (95% CI)
		Positive	Negative	
Benign				
Hyperplastic follicular cell nodule	95 (37)	11	84	88 (80-93)
Hyperplastic Hürthle cell nodule	5 (2)	0	5	100 (57-100)
Follicular adenoma	47 (18)	10	37	79 (65-88)
Hürthle cell adenoma	34 (13)	13	21	62 (45-76)
NIFTP	11 (4)	11	0	100 (74-100)^a^
Malignant				
Papillary thyroid carcinoma	49 (19)	45	4	92 (81-97)
Follicular thyroid carcinoma	4 (2)	3	1	75 (30-99)
Hürthle cell carcinoma	10 (4)	10	0	100 (72-100)
Medullary thyroid carcinoma	1 (0.5)	1	0	100 (5-100)
Metastatic carcinoma^b^	1 (0.5)	1	0	100 (5-100)
Total	257 (100)	105	152	85 (80-89)

Abbreviation: NIFTP, Noninvasive follicular thyroid neoplasm with papillary-like nuclear features.

^a^ Considering positive test result for NIFTP as correct classification.

^b^ Metastatic renal cell carcinoma.

All 11 NIFTP nodules were correctly classified as positive. Among malignant nodules, 45 of 49 (92%) papillary carcinomas, 3 of 4 (75%) follicular carcinomas, and 10 of 10 (100%) Hürthle cell carcinomas were correctly classified as positive. A medullary thyroid carcinoma and a metastatic renal cell carcinoma were also correctly identified.

Of 152 test-negative samples in the study cohort, 5 (3%) were found to be false-negative, all having a GC score of 0. They included samples from 3 Bethesda III cytology nodules, 1 Bethesda IV, and 1 Bethesda V (eTable 5 in the Supplement). Among them, there were 4 papillary carcinomas and 1 minimally invasive follicular carcinoma. These were all T1 or T2 tumors (1-4 cm), intrathyroidal and without vascular invasion or clinical evidence of nodal or distant metastasis.

### Cancer Probability in Specific Genetic Alteration Groups

Among 105 cases with positive GC results, the probability of surgery-requiring disease, defined as cancer or NIFTP, varied depending on specific genetic alterations (Table 3). Two nodules had high-risk *TERT* or *TP53* mutations, of which 1 was a widely invasive follicular carcinoma and the other was a multifocal papillary carcinoma on surgical pathology. Thirteen nodules were positive for either *BRAF *V600E mutation or *NTRK3*, *BRAF*, or *RET* fusion. Histopathologically, these were all cancers, primarily classical papillary carcinomas. Another 60 nodules were positive for *RAS*, *BRAF* K601E, *PTEN*, *IDH2*, or *DICER1* mutation, or *PPARG*-*THADA* fusion. In this group, cancer/NIFTP was found in 37 of 60 (62%) cases and histologically benign nodules in 23 of 60 (38%); most of the cancers were follicular patterned, either follicular variant papillary or follicular carcinomas. Most common mutations (n = 45) involved *RAS* genes, which were associated with a diagnosis of cancer or NIFTP in 72% for *HRAS*, 52% for *NRAS*, and 40% for *KRAS*. Twenty-two nodules were positive for copy number alterations alone. Cancer/NIFTP was found in 13 (59%) of those, and this group was enriched in Hürthle cell carcinoma and follicular variant papillary carcinoma. Finally, 8 samples were positive for gene expression alterations alone (Table 3).

**Table 3.** Probability of Cancer/NIFTP in Specific Molecular Alteration Groups

Group	Molecular Alterations, No.	Prevalence in Test-Positive Samples, No. (%)	Histopathologic Diagnosis, %		Cancer Type/NIFTP (%)
			Cancer/NIFTP	Benign	
High-risk group	*TERT* (and *HRAS*) (1) *TP53* (and *MEN1*) (1)	2 (2)	100	0	Papillary carcinoma (50) Follicular carcinoma (50)
*BRAF*-like group	*BRAF V600E* (9) *NTRK3* fusions (2) *RET* fusions (1) *BRAF* fusions (1)	13 (12)	100	0	Classical papillary carcinoma (92) Follicular variant papillary carcinoma (8)
*RAS*-like group	*NRAS* (21) *HRAS* (18) *KRAS* (5) *EIF1AX* (5) *BRAF K601E* (3) *PTEN* (1) *IDH2* (1) *DICER1* (1) *PPARG* fusions (4) *THADA* fusions (4)	60 (57)	62	38	Follicular variant papillary carcinoma (22) Papillary carcinoma, other variants (17) NIFTP (15) Follicular carcinoma (3) Hürthle cell carcinoma (5)
Copy number alterations group	Copy number alterations	22 (21)	59	41	Hürthle cell carcinoma (32) Follicular variant papillary carcinoma (14) Papillary carcinoma, other variants (9) NIFTP (5)
Gene expression alterations group	Gene expression alterations	8 (8)	75	25	Classical papillary carcinoma (37) NIFTP (13) Other cancers (MTC, mRCC) (25)

Abbreviations: mRCC, metastatic renal cell carcinoma; MTC, medullary thyroid carcinoma; NIFTP, noninvasive follicular thyroid neoplasm with papillary-like nuclear features.

Among 34 test-positive nodules that were pathologically benign on surgery, 23 (67%) were adenomas and 11 (32%) hyperplastic nodules (eTable 6 in the Supplement). However, 32 of 34 (94%) of them showed 1 or more clonal molecular alterations (point mutation, gene fusion, or DNA copy number alterations) present in a large proportion of cells in the nodule, indicating that these nodules represented neoplasia and not hyperplasia.

## Discussion

The main goal of molecular tests for thyroid FNA samples with indeterminate cytology is to correctly identify most of the benign nodules so that diagnostic thyroid surgery can be avoided in these patients. The safety of the approach is predicated by the test ability to detect all types of thyroid tumors and not miss high-risk cancers. The results of this prospective, blinded, multicenter study demonstrate that in nodules with Bethesda III or IV indeterminate cytology, the multigene GC test was highly sensitive (94%) and reasonably specific (82%) for discriminating benign from malignant/NIFTP nodules. With a baseline disease prevalence of 28%, the test yielded an NPV of 97% and a residual cancer risk of 3% in test-negative nodules, which is similar to an average 3.7% cancer risk in nodules diagnosed as benign by FNA cytology.^10^ Although no test has perfect accuracy, it is reassuring that all false-negative cases in the study were low-stage and low-risk cancers by the American Thyroid Association criteria.^3^

**Figure 2.**
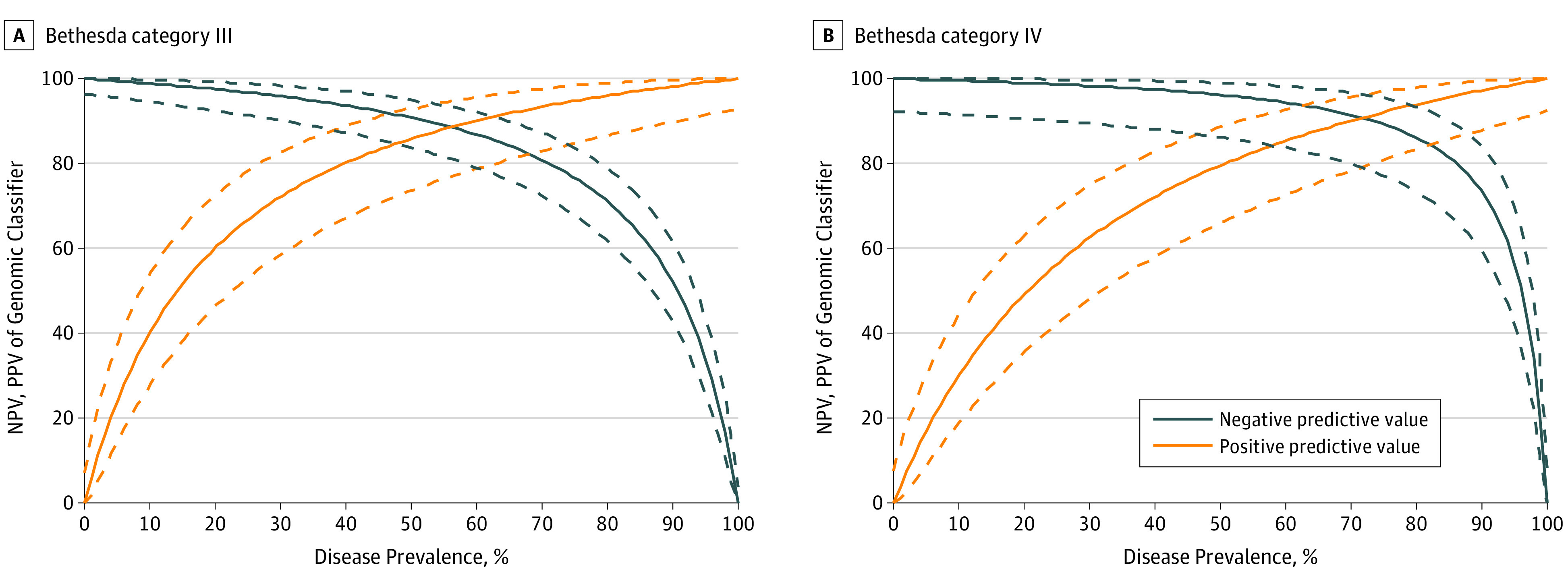
Predicted Performance of Genomic Classifier (GC) Test in Populations With Different Cancer/NIFTP Prevalence Predicted negative predictive value (NPV) (solid blue lines) and positive predictive value (PPV) (solid orange lines) with 95% CIs (dotted lines) based on sensitivity and specificity of the multigene GC test established in this study for Bethesda III and IV cytology thyroid nodules. NIFTP indicates noninvasive follicular thyroid neoplasm with papillary-like nuclear features.

Whereas sensitivity and specificity characterize a test independently of disease prevalence, NPV and PPV depend on the prevalence of disease in the studied population. Based on the fixed sensitivity and specificity, Bayes theorem can predict the test NPV and PPV along the spectrum of disease prevalence.^35^ For the GC test, it predicts a robust NPV of 95% or higher, required to consider nonsurgical treatment by the NCCN guidelines,^36^ up to a disease prevalence of 40% in Bethesda III and 60% in Bethesda IV nodules (Figure 2). This is within the range of cancer/NIFTP probability expected based on the Bethesda reporting system^12,13^ and observed in most clinical studies.^10^

Another commonly used molecular test for thyroid FNA samples is based on measuring expression of multiple genes either by the microarray assay (Gene Expression Classifier; GEC)^27^ or RNA-Seq (Gene Sequencing Classifier; GSC).^37^ In a comparable size validation studies, ThyroSeq GC shows an overall similar sensitivity (94% ThyroSeq vs 90% GEC and 91% GSC) but a specificity of 82% vs 52% in GEC and 68% in GSC (eTable 7 in the Supplement). Furthermore, ThyroSeq GC had a benign or negative call rate of 61% in indeterminate Bethesda III and IV nodules, with 82% of all histologically benign nodules yielding a negative test result. This indicates that ThyroSeq GC can prevent diagnostic surgeries for up to 61% of all of indeterminate Bethesda III to IV cytology nodules and as many as 82% of all benign nodules that yielded indeterminate cytology diagnosis. This should maximize the effect of molecular testing on the avoidance of surgery, reduction of health care costs, and improvement of patient quality of life. This is particularly important during what is widely considered as the era of thyroid cancer overdiagnosis^38^ and overtreatment.^3^

The multigene GC test showed robust performance in detecting all types of thyroid cancer, including Hürthle cell carcinoma. To date, the performance of existing molecular FNA tests in Hürthle cell nodules has been either not specifically reported,^28,29,30^ not validated at all,^31^ or observed to have very low specificity.^39,40^ In this study, all 10 Hürthle cell carcinomas were correctly classified, whereas in all types of Hürthle cell nodules the GC test negative call rate was 53%. This should allow the avoidance of diagnostic surgery in more than half of biopsied Hürthle cell nodules.

Another potential advantage of the GC test is that it provides a molecular profile of the test-positive nodules, which may help clinicians to refine the treatment of patients with Bethesda III, IV, and V nodules and a positive test. Indeed, the finding of *BRAF* V600E and similar alterations as well as high-risk (*TERT*, *TP53*) mutations conferred a 100% probability of cancer in this study, in keeping with previous reports.^41,42,43^ Tumors harboring a *BRAF* V600E mutation are classic papillary carcinoma with a higher rate of regional lymph node metastasis.^3,19^ On the contrary, *RAS* and *RAS-*like alterations were associated with a spectrum of follicular-pattern thyroid tumors, from pathologically benign adenomas to borderline NIFTP and fully invasive cancers, with a roughly 60% probability of cancer/NIFTP. These cancers are frequently encapsulated and if spread, they typically skip regional lymph nodes and metastasize hematogenously.^19^ However, most thyroid cancers driven by single *RAS* and *RAS*-like mutations are minimally invasive and low risk. The histologically benign nodules carrying these mutations are monoclonal tumors, in contrast to polyclonal hyperplastic nodules which are the most common type of benign thyroid nodules. Finally, the GC test correctly classified nodules composed of nonthyroid follicular cells, including medullary carcinoma and a metastatic tumor. This additional information on the test-positive nodules along with clinical factors may help to further individualize patient treatment.

As genetic information becomes available preoperatively, future studies are required to better understand how this information should be integrated with ultrasound and other clinical data to inform more tailored treatment of patients with thyroid nodules and cancers that have different molecular profiles. Furthermore, prospective studies will be needed to determine whether patients with the molecular signature of low-risk cancer or NIFTP can have surgery safely delayed or replaced by medical surveillance, as is currently under consideration for small thyroid cancers.^44,45^

### Limitations

This study has several limitations. By selecting patients based on the Bethesda reporting system for thyroid cytology, the applicability of the findings is limited to practices that use this reporting system. The observed small number of samples from Bethesda V nodules did not allow meaningful test validation in this subset of nodules. By surgically removing nodules with low cancer probability genetic alterations (GC score 1) for final histological diagnosis, the long-term clinical impact of these alterations could not be established. Finally, this study was performed at moderate- to high-volume centers with established thyroid nodule imaging and clinical expertise. Thus, the results may differ for practices that have a different setting and diagnostic approaches to thyroid nodules.

## Conclusions

The study documents a high sensitivity and correspondingly high NPV of the ThyroSeq GC test for Bethesda III and IV indeterminate cytology nodules, which together with high specificity may prevent diagnostic surgeries in the majority of such patients. The availability of detailed genetic information in test-positive cases may help to further inform individualized treatment for these patients after integration with imaging and other clinical information.

## Notes

Supplement. **eMethods 1.** Central pathology review **eMethods 2.** Supplemental statistical analysis **eTable 1.** Agreement between local pathology diagnosis and central pathology review diagnosis **eTable 2.** Distribution of samples and histopathology diagnoses contributed from 10 study sites **eTable 3.** Characteristics of patients and nodules in the study cohort **eTable 4.** Genomic classifier (GC) scores and alteration types detected in samples with negative and positive test results **eTable 5.** Demographic, clinical and pathological characteristics of cases with false negative test result **eTable 6.** Characteristics of 34 cases with false-positive test results **eTable 7.** Study characteristics and performance of ThyroSeq GC and Afirma GEC and GSC in Bethesda III and IV indeterminate cytology thyroid nodules **eFigure 1.** Distribution of GC scores in nodules with different histopathology
