# Supplementary material for: Performance of a Multigene Genomic Classifier in Thyroid Nodules With Indeterminate Cytology: A Prospective Blinded Multicenter Study
Source: JAMA Oncol. 2018 Nov 8;5(2):1–9. doi: 10.1001/jamaoncol.2018.4616 (PMC6439562; doi:10.1001/jamaoncol.2018.4616)
Supplement: Supplement. — eMethods 1. Central pathology review eMethods 2. Supplemental statistical analysis eTable 1. Agreement between local pathology diagnosis and central pathology review diagnosis eTable 2. Distribution of samples and histopathology diagnoses contributed from 10 study sites eTable 3. Characteristics of patients and nodules in the study cohort eTable 4. Genomic classifier (GC) scores and alteration types detected in samples with negative and positive test results eTable 5. Demographic, clinical and pathological characteristics of cases with false negative test result eTable 6. Characteristics of 34 cases with false-positive test results eTable 7. Study characteristics and performance of ThyroSeq GC and Afirma GEC and GSC in Bethesda III and IV indeterminate cytology thyroid nodules eFigure 1. Distribution of GC scores in nodules with different histopathology [file jamaoncol-e184616-s001.pdf]

Supplementary Online Content

Steward DL, Carty SE, Sippel RS, et al. Performance of a multigene genomic classifier in thyroid nodules with indeterminate cytology: a prospective blinded multicenter study [published online November 8, 2018]. *JAMA Oncol*. doi:10.1001/jamaoncol.2018.4616

eMethods 1. Central pathology review
eMethods 2. Supplemental statistical analysis
eTable 1. Agreement between local pathology diagnosis and central pathology review diagnosis
eTable 2. Distribution of samples and histopathology diagnoses contributed from 10 study sites
eTable 3. Characteristics of patients and nodules in the study cohort
eTable 4. Genomic classifier (GC) scores and alteration types detected in samples with negative and positive test results
eTable 5. Demographic, clinical and pathological characteristics of cases with false negative test result
eTable 6. Characteristics of 34 cases with false-positive test results
eTable 7. Study characteristics and performance of ThyroSeq GC and Afirma GEC and GSC in Bethesda III and IV indeterminate cytology thyroid nodules
eFigure 1. Distribution of GC scores in nodules with different histopathology

This supplementary material has been provided by the authors to give readers additional information about their work.

© 2018 American Medical Association. All rights reserved.

Supplementary Online Content

Steward DL; Carty SE, Sippel RS, et al. Performance of ThyroSeq Genomic Classifier in Thyroid Nodules with Indeterminate Cytology: A Prospective Blinded Multicenter Study

eMethods 1. Central pathology review

eMethods 2. Supplemental statistical analysis

eTable 1. Agreement between local pathology diagnosis and central pathology review diagnosis

eTable 2. Distribution of samples and histopathology diagnoses contributed from 10 study sites

eTable 3. Characteristics of patients and nodules in the study cohort

eTable 4. Genomic classifier (GC) scores and alteration types detected in samples with negative and positive test results

eTable 5. Demographic, clinical and pathological characteristics of cases with false negative test result

eTable 6. Characteristics of 34 cases with false-positive test results

eTable 7. Study characteristics and performance of ThyroSeq GC and Afirma GEC and GSC in Bethesda III and IV indeterminate cytology thyroid nodules

eFigure 1. Distribution of GC scores in nodules with different histopathology

© 2018 American Medical Association. All rights reserved.

eMethods 1. Central Pathology Review

Central pathology review was performed by a panel of three expert thyroid pathologists (Z.W.B., R.V.L., R.R.S.) using digitalized images from representative histological slides of each nodule. The reviewers were blinded to the results of local pathology and each other's diagnosis. Cases with discrepant diagnoses issued by the panel pathologists were re-reviewed at a teleconference, and a consensus diagnosis was reached in all cases. For nodules for which digitalized images were not available for review (n=12, 4%), local pathology diagnosis was accepted as final diagnosis.

© 2018 American Medical Association. All rights reserved.

eMethods 2. Supplemental statistical analysis

Sample size justification

We planned to enroll 400 subjects for this study. Our objective was to demonstrate an increase in NPV from the current aggregate estimate of 89% to a targeted NPV of 95% with a maximum 95% confidence interval width of +/- 5% for the ThyroSeq genomic classifier. We assumed the classifier would be capable of 84% sensitivity, an improvement over the 63% of the older 7 gene panel classifier. Assuming a prevalence of malignant disease (24%) and the specificity of 91%, 84% sensitivity will provide the desired 95% NPV. A minimum of 200 patients with indeterminate cytology and surgical pathology outcome will be required to guarantee the desired 95% confidence interval on NPV with a lower boundary of 90%. Allowing for lower than anticipated disease prevalence we elected to enroll 400 patients from the original participating 8 sites (~50 from each site).

The programming code used to generate results

```
#---------------- Table 1 -----------------------

#--------- Performance of +/- 1.5 cutoff, separately for Bethesda Category and diag2/diag3
table(Score > 1.5, diag2)
# fix collating order for function class.oc()
A2$Sc <- ifelse(Score > 1.5, " pos", "neg")
# create ordered factors Diag2 and Diag3, for use of my function class.oc()
A2$Diag2 <- ordered(diag2, levels = c("Malignant", "Benign"))

attach(A2) # version with cutoff Sc



# All
table(Sc, Diag2)
class.oc(table(Sc, Diag2))
# Bethesda category 3
table(Sc[Bethesda.Category == 3], Diag2[Bethesda.Category == 3])
class.oc(table(Sc[Bethesda.Category == 3], Diag2[Bethesda.Category == 3]))
# Bethesda category 4
table(Sc[Bethesda.Category == 4], Diag2[Bethesda.Category == 4])
class.oc(table(Sc[Bethesda.Category == 4], Diag2[Bethesda.Category == 4]))
# Bethesda categories 3 & 4
table(Sc[Bethesda.Category %in% c(3,4)], Diag2[Bethesda.Category %in% c(3,4)])
class.oc(table(Sc[Bethesda.Category %in% c(3,4)], Diag2[Bethesda.Category %in% c(3,4)]))

#--------------- Figure 2 ------------------------
plot.PV <- function(p, tpf, fpf, n, m, refline = NULL, xlab = "Disease Prevalence (%)", x.leg = 43, y.leg = 40, legend = T,...) {
  # p = prevalene, n = # negative tests, m = # positive tests
   NPV <- ((1-p) * (1-fpf)) / (((1-p) * (1-fpf)) + (p * (1-tpf)))
   P2PV <- (p * tpf) / ((p * tpf) + ((1-p) * fpf))
   ci.neg <- binconf(NPV * n,n)
   ci.pos <- binconf(PPV * m,m)
   Z.neg <- data.frame(p, NPV, ci.neg)
   Z.pos<- data.frame(p, PPV, ci.pos)
```

© 2018 American Medical Association. All rights reserved.

```
   # for plotting  change p and PV's to a percent
   plot(Z.neg$p * 100, Z.neg$NPV * 100,  type = "l", pty = "n", xaxt = "n", yaxt = "n", xlab = xlab,...)
   axis(1, at = seq(0,100,10))
   axis(2, at = seq(0,100,10), las  = 1)
   lines(Z.neg$p * 100, Z.neg$NPV * 100, lty = 1, col= "blue", lwd = 3)
   lines(Z.neg$p * 100, Z.neg$Lower * 100, lty = 2, col = "blue", lwd = 2)
   lines(Z.neg$p * 100, Z.neg$Uppe * 100, lty = 2, col = "blue", lwd = 2)

   lines(Z.pos$p * 100, Z.pos$PPV * 100, lty = 1, col = "red3", lwd = 3)
   lines(Z.pos$p * 100, Z.pos$Lower * 100, lty = 2, col = "red3", lwd = 2)
   lines(Z.pos$p * 100, Z.pos$Upper * 100, lty = 2, col = "red3", lwd = 2)
    abline(h = refline)
    #grid()
    abline(h = seq(0,100, by = 10), v = seq(0,100, by = 10), lty = "dotted", col = "lightgray")
        if(legend) legend(x.leg, y.leg,  c("NPV", "PPV"), lty = 1, lwd = 2, col = c("blue","red3" ))
  invisible(data.frame(Z.neg, Z.pos))
}


# plot only BC3
# Bethesda category 3
table(Sc[Bethesda.Category == 3], Diag2[Bethesda.Category == 3])
class.oc(table(Sc[Bethesda.Category == 3], Diag2[Bethesda.Category == 3]))
#num pos tests = 50; num neg tests = 104, sen = .914, spec = .849
par(pin = c(5,3), cex.axis = 1.15, cex.lab = 1.25)
aa <- plot.PV(p = seq(0, 1, length = 100), tpf = .914, fpf = 1 - .849, n = 104, m = 50, refline = NA,
  ylab = "NPV, PPV of ThyroSeq GC", main = "Bethesda Category III", legend = F)

# plot only BC4
# Bethesda category 4
table(Sc[Bethesda.Category == 4], Diag2[Bethesda.Category == 4])
class.oc(table(Sc[Bethesda.Category == 4], Diag2[Bethesda.Category == 4]))
#num pos tests = 47; num neg tests = 46, sen = .970, spec = .750
aa <- plot.PV(p = seq(0, 1, length = 100), tpf = .970, fpf = 1 - .750, n = 46, m = 47, refline = NA,
  ylab = "NPV, PPV of ThyroSeq GC", main = "Bethesda Category IV", legend = F)

# plot only Bc3 + BC4
# Bethesda categories 3 & 4
table(Sc[Bethesda.Category %in% c(3,4)], Diag2[Bethesda.Category %in% c(3,4)])
class.oc(table(Sc[Bethesda.Category %in% c(3,4)], Diag2[Bethesda.Category %in% c(3,4)]))
#num pos tests = 97; num neg tests = 150, sen = .941, spec = .816 (slightly different from above?
aa <- plot.PV(p = seq(0, 1, length = 100), tpf = .941, fpf = 1 - .816, n = 150, m = 97, refline = NA,
  ylab = "NPV, PPV of ThyroSeq v3 Test", main = "Bethesda Categories III & IV", legend = F)

#------------- eTable 1 ---------------
#------- sample equivalency ---------------
# The final validation set is a subset of a larger sample. Is the final selection difference from those not selected?
# compare age,gender and (mean)nodule size and number of nodules between
# all with indeterminate cytology (318) but excluded (86) and the 232 finalists

table(D$Exclusion)
# remove those excluded for not having indeterminate cytology (code 0)
ex <- subset(D, Exclusion %in% c(1,2,3,4,6,7))
inc <- subset(D, Exclusion == 5)
B <- data.frame(rbind(ex, inc))
B$group <- c(rep("ex", nrow(ex)), rep("inc",nrow(inc)))
```

© 2018 American Medical Association. All rights reserved.

```
table(B$group)


test2(B$Age, B$group, T)
tab.test(B$Gender, B$group)
test2(B$mean.size, B$group, T)
test2(B$num.nodules, B$group, T)


#------------ eFigure 1 ------------------

cd <- MC$Consensus.Dx
cd2 <- cd
cd2[cd == "PTC, EFV"] <- "PTC"
cd2[cd == "PTC, FV"] <- "PTC"
cd2[cd == "OFTC"] <- "HCC"
cd2[cd == "MRCC"] <- "mRCC"
cd2[cd == "OHN"] <- "HN,HC"
cd2[cd == "OFA"] <- "HCA"
table(MC$Consensus.Dx, cd2)
cd3 <- ordered(cd2, levels = c("HN", "HN,HC", "FA", "HCA", "NIFTP", "PTC", "FTC", "HCC", "MTC",
"mRCC"))
table(MC$Consensus.Dx, cd3)

# calculate "percent of each score"
# create a special data frame for this
t10 <- table(cd3, Score)
tot <- apply(t10, 1, sum)
t11 <- apply(t10, 2, function (x) x / tot * 100)
t12 <- data.frame(row.names(t11), t11)
names(t12)[1] <- "cd3"

MC4 <- reshape(t12, direction = "long", varying = 2:8, idvar = "cd3", v.names = "pct", timevar = "Score", times =
0:6)
MC4$cd4 <- ordered(MC4$cd3, levels = c("HN", "HN,HC", "FA", "HCA", "NIFTP", "PTC", "FTC", "HCC",
"MTC", "mRCC"))

# using a custom pallette
# get two shades of green for scores (0,1); get five shades of red for scores 2 - 6
greens <- rgb(c(.9, .7), red = 0, blue = 0)
#reds <- rgb(c(1, .85, .7, .55, .4), green = 0, blue = 0)
reds <- rgb(c(1,.9, .8, .7,.6), green = 0, blue = 0)
custom.colors <- c(greens, reds)
showpanel(custom.colors)

# finally there is a request to remove the last 2 categories that each have only 1 specimen
MC4.temp <- subset(MC4,  cd4 != "MTC" & cd4 != "mRCC")

ggplot(MC4.temp, aes(x = cd4, y = pct, fill = as.factor(Score))) + geom_bar(stat = "identity")+ labs(fill = "Score") +
  xlab("Pathology Diagnosis") + ylab("Percentage") +
 scale_fill_manual(values = custom.colors)+ guides(fill = guide_legend(reverse = T)) +
  theme(axis.text.x = element_text(size = rel(1.25))) + theme(axis.text.y = element_text(size = rel(1.25))) +
  theme(axis.title.x = element_text(size = rel(1.25))) + theme(axis.title.y = element_text(size = rel(1.25))) +
  theme(legend.text = element_text(size = rel(1.25))) + theme(legend.title = element_text(size = rel(1.25)))

#-------------------------------------------------------------------
```

© 2018 American Medical Association. All rights reserved.

eTable 1. Agreement between local pathology diagnosis and central pathology review diagnosis

Agreement/disagreement	Number of cases	%
Complete agreement	209	76.3
Minor disagreement[a]	45	16.4
Moderate disagreement[b]	11	4.0
Major disagreement[c]	9	3.3
Total available for central review	274	100

[a] Minor disagreement: disagreement between subtypes of benign or malignant nodules
[b] Moderate disagreement: benign vs NIFTP or NIFTP vs malignant
[c] Major disagreement: benign vs malignant

© 2018 American Medical Association. All rights reserved.

eTable 2. Distribution of samples and histopathology diagnoses contributed from 10 study sites

Site number	Total samples	Histopathology diagnosis*			Cancer+NIFTP prevalence
		Benign*	Malignant	NIFTP	
**1**	91	73	16	2	20%
**2**	88	66	18	4	25%
**3**	21	12	8	1	43%
**4**	20	8	11	1	60%
**5**	19	15	3	1	21%
**6**	13	7	6	0	46%
**7**	11	6	4	1	45%
**8**	11	10	1	0	9%
**9**	6	4	1	1	33%
**10**	6	5	1	0	17%
Total	286	206	69	11	28%

*Based on central pathology review

© 2018 American Medical Association. All rights reserved.

eTable 3. Characteristics of patients and nodules with indeterminate cytology in the excluded and evaluated cohorts

**Characteristic**	All Cases with Indeterminate Cytology	Indeterminate Cytology and Excluded	Final Evaluation Cohort	Test of Equality*
**Total no**				
Patients	318	86	232	N/A
Nodules	350	93	257	
**Gender**				
Female	251 (79%)	65 (76%)	186 (80%)	.44
Male	67 (21%)	21 (24%)	46 (20%)	
**Age**				
Median	54	54.5	53	.01
IQR	43 – 62	43 - 66	42 – 61	
**Nodule Size by Ultrasound, mean (cm)**	2.42	2.5	2.4	.12
**Number of Nodules per Patient, mean**	1.24	1.23	1.25	.72

* Fisher's Exact test for age, Wilcoxon test for others, all tests are two tailed.

© 2018 American Medical Association. All rights reserved.

eTable 4. Genomic classifier (GC) scores and alteration types detected in samples with negative and positive test results

Test result	GC score	Number of cases	SNV/indels	Gene fusions	CNA	GEA
Negative (n=152)	0	131	0	0	0	0
	1	21	14	0	6	1
Positive (n=105)	2	57	26	2	23	7
	3	2	2	0	0	0
	4	30	23	7	5	25
	5	5	4	1	4	4
	6	11	9	2	11	9

SNV, single nucleotide variations; Indels, insertions or deletions; CNA, copy number alterations; GEA, gene expression alterations

© 2018 American Medical Association. All rights reserved.

eTable 5. Demographic, clinical and pathological characteristics of cases with false negative test result

Patient	Age	Sex	Nodule Size by US (cm)	Cytology Diagnosis	Contributing Pathology Diagnosis	Final Pathology Diagnosis	Vascular invasion	Extra-thyroidal extension
1	52	F	4.0	Bethesda III	FTC	PTC	No	No
2	65	M	3.0	Bethesda III	FA	PTC, EFV	No	No
3	57	F	3.2	Bethesda III	FTC	FTC	No	No
4	29	F	4.0	Bethesda IV	PTC	PTC	No	No
5	56	F	1.1	Bethesda V	PTC	PTC	No	No

FTC, Follicular thyroid carcinoma; FA, follicular adenoma; PTC, papillary thyroid carcinoma; PTC, EFV, encapsulated follicular variant, US, ultrasound

© 2018 American Medical Association. All rights reserved.

eTable 6. Characteristics of 34 cases with false-positive test results

**Cytology diagnosis**	**n (%)**
Bethesda III	18 (53%)
Bethesda IV	15 (44%)
Bethesda V	1 (3%)
**Pathology findings**	
HCA	13 (38%)
FA	10 (29%)
HN	11 (32%)
**Molecular findings**	
Point mutations	21 (62%)
RAS	18*
BRAF K601E	1
EIF1AX	1
DICER1	1
Fusions	2 (6%)
THADA/IGF2BP3	1
PAX8/PPARG	1
Copy number alterations (CNA)	15 (44%)
Gene expression alterations (GEA)	14 (41%)
Total cases with one or more clonal genetic alteration*	***32 (94%)***

*Including 10 *NRAS*, 5 *HRAS*, and 3 *KRAS*
**Including point mutations, gene fusions, and copy number alterations; excluding gene expression alterations.
HCA, Hürthle cell adenoma; FA, follicular adenoma; HN, hyperplastic follicular cell nodules and hyperplastic Hürthle cell nodules

© 2018 American Medical Association. All rights reserved.

eTable 7. Study characteristics and performance of ThyroSeq GC and Afirma GEC and GSC in Bethesda III and IV indeterminate cytology thyroid nodules

	ThyroSeq GC[1]	***Afirma GEC***[2]	***Afirma GSC***[3]
Study type	Multicenter, prospective, double-blind	Multicenter, prospective, double-blind	Multicenter, retrospective, double-blind
Total number, samples	247	210	191
Total number, patients	223	199	183
Age, mean (range), years	51.7 (18-90)	51.2 (22-85)	51.6 (18-90)
Female, %	80	77	78
Nodule size by ultrasound, median (range), cm	2.1 (0.5-7)	2.5 (1-9.1)	2.6 (1.0-9.1)
Disease prevalence, %	27.5	24.3	23.7
Sensitivity, % (95%CI)	94.1 (86-98)	90.2 (79-97)	91.1 (79-98)
Specificity, % (95%CI)	81.6 (75-87)	51.6 (44-60)	68.3 (60-76)
NPV	97.3 (93-99)	94.3 (88-97)	96.1 (90-99)
PPV	65.9 (56-75)	37.4 (33-42)	47.1 (36-58)
Benign call rate	61%	41%	54%
Avoidable surgeries for histologically benign nodules with indeterminate cytology	82%	52%	68%

[1]Current study
[2]Alexander EK, Kennedy GC, Baloch ZW, et al. Preoperative diagnosis of benign thyroid nodules with indeterminate cytology. The New England journal of medicine. 2012;367(8):705-715.
[3]Patel KN, Angell TE, Babiarz J, et al. Performance of a Genomic Sequencing Classifier for the Preoperative Diagnosis of Cytologically Indeterminate Thyroid Nodules. *JAMA Surg*. 2018. Published online May 23, 2018. doi:10.1001/jamasurg.2018.1153

© 2018 American Medical Association. All rights reserved.

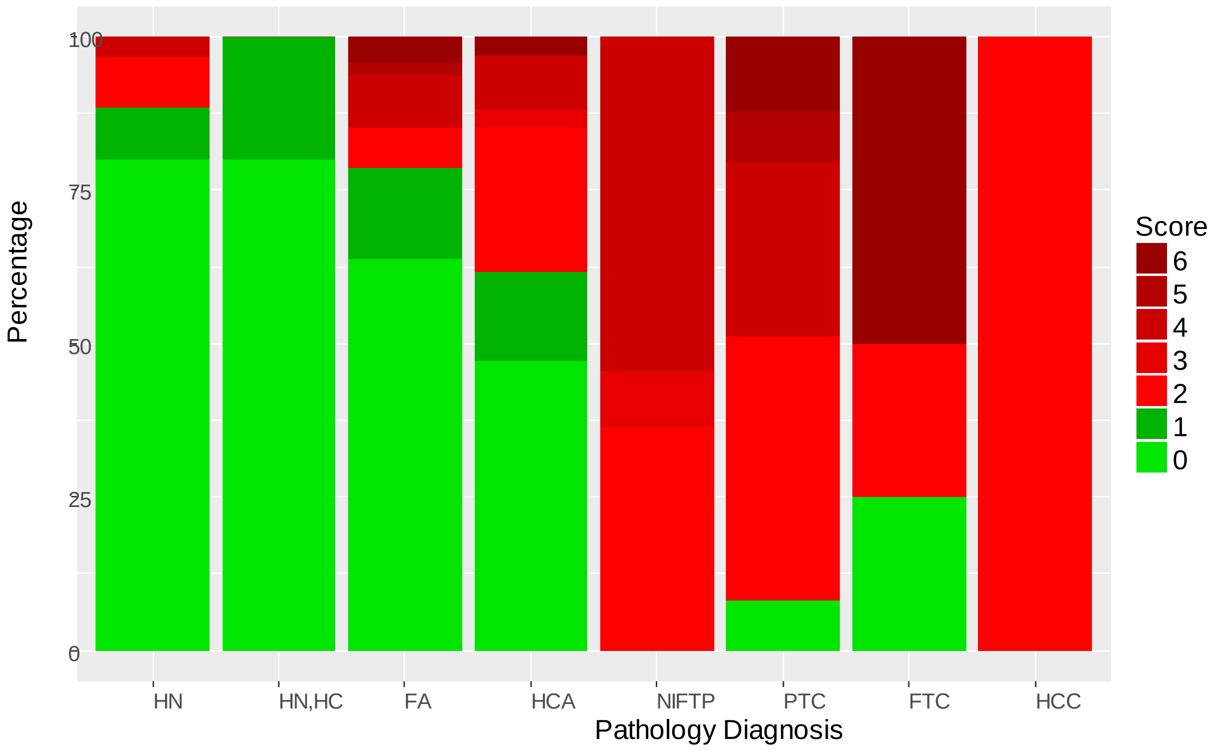

HN, hyperplastic follicular cell nodules; HN,HC, hyperplastic Hürthle cell nodules; FA, follicular adenoma; HCA, Hürthle cell adenoma; NIFTP, non-invasive follicular thyroid neoplasm with papillary-like nuclear features; PTC, papillary thyroid carcinoma; FTC, follicular thyroid carcinoma; HCC, Hürthle cell carcinoma; GC, genomic classifier.

eFigure 1. Distribution of GC scores in nodules with different histopathology.

© 2018 American Medical Association. All rights reserved.
